# The Porcine Deltacoronavirus Replication Organelle Comprises Double-Membrane Vesicles and Zippered Endoplasmic Reticulum with Double-Membrane Spherules

**DOI:** 10.3390/v11111030

**Published:** 2019-11-05

**Authors:** Nicole Doyle, Philippa C. Hawes, Jennifer Simpson, Lorin H. Adams, Helena J. Maier

**Affiliations:** The Pirbright Institute, Ash Road, Woking, Surrey GU24 0NF, UK; nicole.doyle@pirbright.ac.uk (N.D.); pippa.hawes@pirbright.ac.uk (P.C.H.); jennifer.simpson@pirbright.ac.uk (J.S.); lorin.adams@pirbright.ac.uk (L.H.A.)

**Keywords:** porcine deltacoronavirus, coronavirus, replication organelle, double-membrane vesicle, DMV, zippered ER, spherule, double-membrane spherule

## Abstract

Porcine deltacoronavirus (PDCoV) was first identified in Hong Kong in 2012 from samples taken from pigs in 2009. PDCoV was subsequently identified in the USA in 2014 in pigs with a history of severe diarrhea. The virus has now been detected in pigs in several countries around the world. Following the development of tissue culture adapted strains of PDCoV, it is now possible to address questions regarding virus–host cell interactions for this genera of coronavirus. Here, we presented a detailed study of PDCoV-induced replication organelles. All positive-strand RNA viruses induce the rearrangement of cellular membranes during virus replication to support viral RNA synthesis, forming the replication organelle. Replication organelles for the *Alpha-*, *Beta*-, and *Gammacoronavirus* genera have been characterized. All coronavirus genera induced the formation of double-membrane vesicles (DMVs). In addition, *Alpha*- and *Betacoronaviruses* induce the formation of convoluted membranes, while *Gammacoronaviruses* induce the formation of zippered endoplasmic reticulum (ER) with tethered double-membrane spherules. However, the structures induced by *Deltacoronaviruses*, particularly the presence of convoluted membranes or double-membrane spherules, are unknown. Initially, the dynamics of PDCoV strain OH-FD22 replication were assessed with the onset of viral RNA synthesis, protein synthesis, and progeny particle release determined. Subsequently, virus-induced membrane rearrangements were identified in infected cells by electron microscopy. As has been observed for all other coronaviruses studied to date, PDCoV replication was found to induce the formation of double-membrane vesicles. Significantly, however, PDCoV replication was also found to induce the formation of regions of zippered endoplasmic reticulum, small associated tethered vesicles, and double-membrane spherules. These structures strongly resemble the replication organelle induced by avian *Gammacoronavirus* infectious bronchitis virus.

## 1. Introduction

All positive strand RNA (+RNA) viruses studied to date have rearranged the membranes of their host cell to create the virus replication organelle (RO). For several viruses, ROs have been proven to be the site of viral RNA synthesis [1,2,3,4]. As such, ROs are thought to facilitate the coordination of the processes involved in viral RNA synthesis, as well as provide an enclosed environment to protect viral RNA from detection by the host cell. This helps to prevent degradation of viral RNA by host enzymes and, importantly, prevent activation of cellular intrinsic antiviral signaling pathways. Therefore, RO formation and function are critical steps in the replication cycle of all +RNA viruses. The membrane origin, shape, and structure of ROs varies between different virus families, but there are common themes in the types of structure produced. Several viruses, including enteroviruses, arteriviruses, toroviruses, hepatitis C virus, and foot and mouth disease virus, induce the formation of double-membrane vesicles (DMVs), often along with single-membrane vesicles and tubules or paired membranes [5,6,7,8,9,10]. The second type of structure which are commonly induced are spherules or invaginated vesicles, which are formed by alphaviruses, flaviviruses, bromoviruses, and nodaviruses [3,4,11,12,13,14,15]. These are small single-membrane vesicles which are pinched out from a cellular membrane, but they remain tethered to the membrane and possess a channel connecting the interior of the spherule with the cytoplasm.

An important family of +RNA viruses are the coronaviruses. Coronaviruses infect a wide range of species, including swine, cattle, poultry, dogs, cats, horses, and humans, causing economically damaging diseases impacting livestock farming industries, significant companion animal morbidity, and important human illnesses. In the last 17 years, two novel coronaviruses, severe acute respiratory syndrome coronavirus (SARS-CoV) and Middle East respiratory syndrome coronavirus (MERS-CoV), have emerged into the human population from zoonotic sources (bats via civets and camels, respectively) and have caused significant illnesses with high mortality rates [16,17]. This, along with the recent emergence of swine acute diarrhea syndrome (SADS)-CoV in pigs arising after a species jump [18,19], highlights the importance of this family of viruses as a threat to human health with the potential for further emergence of new pathogenic viruses. There are four genera within the coronavirus family, *Alpha*-, *Beta*-, *Gamma*-, and *Deltacoronaviruses,* and the ROs of viruses within these genera have been characterized. All coronaviruses induce the formation of DMVs. In addition, there are rearrangements of the endoplasmic reticulum (ER) to form either a branching network of membranes, referred to as convoluted membranes (CM) found in *Alpha-* and *Betacoronavirus* infected cells [20,21,22,23], or paired ER membranes, referred to as zippered ER in *Gammacoronavirus* infectious bronchitis virus (IBV) infected cells identified in our previous work [24,25]. Small double-membrane spherules, not seen previously in cells infected with other coronaviruses, are associated with the zippered ER in IBV-infected cells are.

*Deltacoronaviruses* were first characterized as a new coronavirus genus in 2011. The majority of members of this genus infect avian species and have been identified only through sequencing the viral genome. Therefore, in the absence of viral isolates able to replicate in cell culture, studying the virus–host interactions of this genus of coronaviruses has not been possible. However, porcine deltacoronavirus (PDCoV) was identified in Hong Kong in 2012 [26], and subsequently from pigs in the USA and other countries [27,28,29,30,31]. The virus causes an acute gastrointestinal infection with severe diarrhea, vomiting, and atrophic enteritis [32]. Importantly, cell culture adapted strains of PDCoV have now been developed [33,34,35], allowing the characterization of how this genus of coronaviruses interacts with its host cell, including a characterization of the *Deltacoronavirus* RO. In a recent publication, Qin et. al. confirmed the presence of DMVs in PDCoV infected cells [36]. However, neither CM nor zippered ER and spherules were identified. Here, we characterized PDCoV stain OH-FD22 [34] replication in porcine LLC-PK1 cells, including a detailed characterization of ROs.

## 2. Materials and Methods

### 2.1. Cells and Virus

Porcine LLC-PK1 cells (ATCC CL-101) [37] were maintained in Dulbecco’s Modified Eagle’s Medium (DMEM; Sigma Aldrich, Gillingham, UK) supplemented with 10% FCS (Sigma Aldrich). Porcine deltacoronavirus OH-FD22 was kindly provided by Prof. Linda Saif, The Ohio State University [32,34]. Viral infection of LLC-PK1 cells was performed in EMEM supplemented with 1% HEPES, 1% NEAA, and 1% antibiotic-antimycotic with 2.5–10 μg/mL trypsin. When approximately 80% CPE was visible, cells and culture media were harvested, freeze/thawed twice, and cell debris were pelleted. Viral stocks were titrated by tissue culture infectious dose 50 (TCID_50_).

### 2.2. Reverse Transcription and Quantitative Polymerase Chain Reaction

LLC-PK1 cells were seeded into six-well plates (6 × 10^5^ cells/well) 24 h prior to use and were used at 70–90% confluence. Cells were mock-infected or infected with PDCoV (10^3.8^ TCID_50_ units/well). At the indicated timepoints, cells were scraped into phosphate-buffered saline (PBS) and pelleted. Cell pellets were lysed in RLT buffer (Qiagen, Hilden, Germany) and RNA extracted using an RNeasy kit following the manufacturer’s instructions. RNA was eluted into 50 µL RNAse-free water. Complementary DNA was generated using superscript IV (Invitrogen, Renfrew, UK) following the manufacturer’s instructions and using 300 ng RNA and a random primer. Quantitative polymerase chain reaction (PCR) was performed using Taqman Fast Universal 2× Master Mix (Invitrogen) including 125 nM probe, 500 nM primers, and 2 µL cDNA in a 10 µL reaction. Primer and probe sequences within the PDCoV M gene have been described previously [34,38]. RNA levels in virus -infected samples were normalized to mock and subsequent absolute quantitation of cDNA copies was performed using a standard curve generated using a PCR product from the *M* gene covering the qPCR amplified fragment [34] (Supplementary Figure S1).

### 2.3. Western Blot

LLC-PK1 cells were seeded into six-well plates (6 × 10^5^ cells/well) 24 h prior to use and were used at 70–90% confluence. Cells were mock-infected or infected with PDCoV (10^3.8^ TCID_50_ units/well). At the indicated time points, cells were scraped into PBS and pelleted. The cell pellet was lysed in 1× sample buffer (Biorad Laboratories, Watford, UK) containing β-mercaptoethanol, sonicated for 2 min (70% amplitude) and heated to 95 °C for 3 min. Proteins were separated by SDS-PAGE and transferred onto nitrocellulose membrane. After blocking in 5% milk in PBS-Tween 20 (PBS-T), membranes were incubated with primary antibodies to detect PDCoV nucleoprotein (N) (Alpha Diagnostic International) and actin (Abcam, Cambridge, UK) diluted in blocking buffer. After washing in PBS-T, membranes were incubated with IRDye labelled secondary antibody (LI-COR, Cambridge, UK) diluted in blocking buffer. After further washes, membranes were imaged using an Odyssey CLx Infrared imaging system (LI-COR).

### 2.4. Virus Growth Curve and Titration by TCID_50_

LLC-PK1 cells were seeded into six-well plates (6 × 10^5^ cells/well) 24 h prior to use and were used at 70–90% confluence. Cells were mock-infected or infected with PDCoV (10^3.8^ TCID_50_ units/well). At the indicated timepoints, culture media was harvested and stored at −80 °C. Virus was titrated by TCID_50_. Briefly, cells seeded into 96-well plates were infected with a two-fold serial dilution series of virus. Cells positive and negative for cytopathic effect (CPE) were scored 5 d post-infection, and viral titer was calculated using the Reed and Muench method.

### 2.5. Immunofluorescence

LLC-PK1 cells were seeded into 24-well plates on coverslips (2 × 10^5^ cells/well) 24 h prior to use and were used at 70–90% confluence. Cells were mock-infected or infected with PDCoV (10^3.3^ TCID_50_ units/well). At the indicated timepoints, cells were fixed with 4% paraformaldehyde in PBS and permeabilized with 0.1% triton X-100 in PBS. After blocking in 0.5% bovine serum albumin (BSA; Sigma Aldrich) in PBS, cells were labelled with primary antibodies specific for PDCoV N and dsRNA (J2, English and Scientific Consulting Kft., Budapest, Hungary). Cells were washed in PBS and labelled with Alexa Fluor 488 or 568 conjugated secondary antibodies (Invitrogen) diluted in blocking buffer. After further washing, nuclei were stained using 4’,6-diamidino-2-phenylindole (DAPI), mounted onto glass slides using Vectashield (Vector Labs, Peterborough, UK) and sealed using nail varnish. Cells were visualized using a Leica SP5 or SP8 confocal microscope (Leica Microsystems, Milton Keynes, UK). Images were compiled using Adobe Photoshop.

For the detection of nascent viral RNA, 30 min prior to fixation, cells were treated with 2 mM BrU (Sigma Aldrich) and either 15 µM ActD (Sigma Aldrich) or DMSO as a vehicle control. Cells were fixed and labelled as above with the following modifications: 0.1% fish skin gelatin (Sigma Aldrich) in PBS was used as the blocking buffer, and all steps following fixation were performed in an RNAse-free environment with the inclusion of RNAsin (Promega, Southampton, UK) in all buffers to prevent loss of the BrU signal [39]. The primary antibody specific for BrdU, which also recognizes BrU, was purchased from Roche. Primary antibody-recognizing ER chaperone, ERp57, was kindly provided by Dr Chris Netherton, The Pirbright Institute [40]. For co-labelling of PDCoV N protein with BrU, anti-N was conjugated to Alexa Fluor 647 using a Xenon antibody labelling kit (Invitrogen) following the manufacturer’s instructions.

### 2.6. Transmission Electron Microscopy

LLC-PK1 cells were seeded into 24-well plates on Thermanox coverslips (Fisher Scientific, Loughborough, UK; 2 × 10^5^ cells /well) 24 h prior to use and were used at 70–90% confluence. Cells were mock-infected or infected with PDCoV (10^3.3^ TCID_50_ units/well). At the indicated timepoints, cells were fixed in 2% glutaraldehyde for 1 h. Cells were incubated for 1 h in 1% aqueous osmium tetroxide solution then dehydrated in increasing concentrations of ethanol. Following embedding in Agar 100 resin (Agar Scientific Ltd, Stansted, UK) and polymerization overnight, 80 nm thick sections were cut, collected on hexagonal 200 thin bar copper grids, and stained with lead citrate and 2% uranyl acetate. Data were collected using a FEI Tecnai 12 TEM (FEI, Cambridge, UK) at 100 kV with a TVIPS F214 digital camera. Images were compiled using Adobe Photoshop.

## 3. Results

### 3.1. Kinetics of Porcine deltacoronavirus Replication in LLC-PK1 Cells

**Figure 1:**
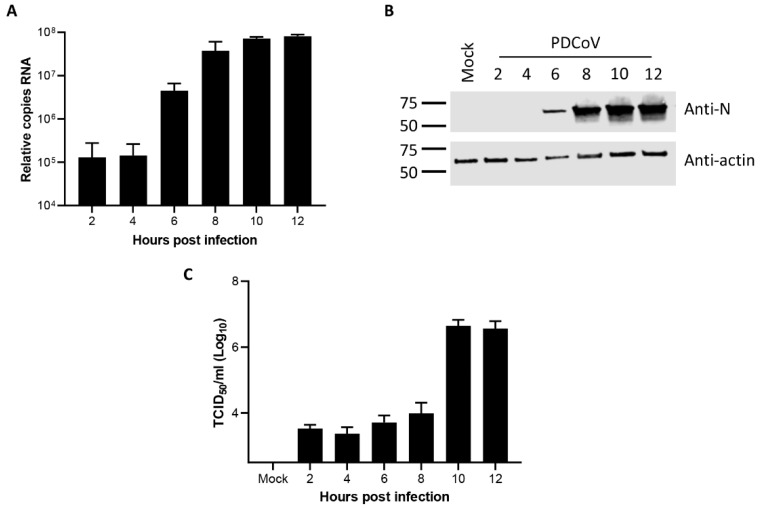
Dynamics of porcine deltacoronavirus (PDCoV) OH-FD22 replication in LLC-PK1 cells. (**A**) LLC-PK1 cells were infected with PDCoV and at the indicated timepoints, RNA was harvested and reverse transcribed to cDNA. The copy number of cDNA was quantified by quantitative polymerase chain reaction (qPCR) using a standard curve and normalized to mock-infected cells. Mean and standard deviation of three independent replicates are shown. (**B**) LLC-PK1 cells were PDCoV-infected or mock-infected. Total cell lysate was harvested at the stated timepoints and viral nucleoprotein detected (Anti-N) by western blot. Actin (Anti-actin) was used as a loading control. Molecular weight markers are shown. Blot representative of three independent repeats. (**C**) LLC-PK1 cells were infected with PDCoV and at the indicated timepoints and cell culture media was harvested. The titer of progeny virus was determined by tissue culture infectious dose 50 (TCID_50_). Mean and standard deviation from three independent replicates are shown.

Initially, the dynamics of PDCoV OH-FD22 replication in LLC-PK1 cells were determined to both confirm successful completion of the virus replication cycle in these cells and to provide a context for subsequent experiments characterizing viral ROs. The accumulation of viral RNA was first measured by real-time quantitative PCR (RT-qPCR) using a primer-probe set specific for the *M* gene, which detected viral genomic RNA, as well as all subgenomic RNAs containing the *M* gene sequence. Cells were infected or mock-infected, and at the indicated timepoints, RNA was extracted, and RT-qPCR was performed (Figure 1A). Viral RNA was detected from the earliest timepoint tested (2 h post-infection (hpi)) but remained constant until 4 hpi. By 6 hpi, the total level of RNA had increased, with further increases detected until 10 hpi. No significant increase was observed between 10 hpi and 12 hpi. This indicates the synthesis of new viral RNA from 6 hpi onward.

Following characterization of total viral RNA levels within infected cells, the levels of viral protein were measured. Cells were infected or mock-infected, and total cell lysates were harvested at the indicated timepoints. The presence of the PDCoV N protein was then determined by western blot (Figure 1B). A band corresponding in size to PDCoV N was detected in all virus-infected samples but was not detected in the mock sample. The N protein was detectable from 6 hpi, with the level increasing by 8 hpi and then remaining constant through to 12 hpi. This confirms the synthesis of new viral proteins from 6 hpi onward.

Finally, the release of progeny virions from PDCoV infected cells was measured. Cells were infected with PDCoV and the cell culture medium was harvested at the indicated timepoints. The amount of virus contained in these samples was measured by TCID_50_ (Figure 1C). Virus were detected in all samples, with a clear increase in the amount of virus present in the cell culture media from 10 hpi, indicating new virions are released from the host cell by this timepoint post-infection. Taken together, this data confirm that PDCoV completes the full virus replication cycle in LLC-PK1 cells with synthesis of new viral RNA and protein occurring between 4 hpi and 6 hpi and release of progeny virions between 8 hpi and 10 hpi.

### 3.2. Visualizing Porcine deltacoronavirus RNA Synthesis 

**Figure 2:**
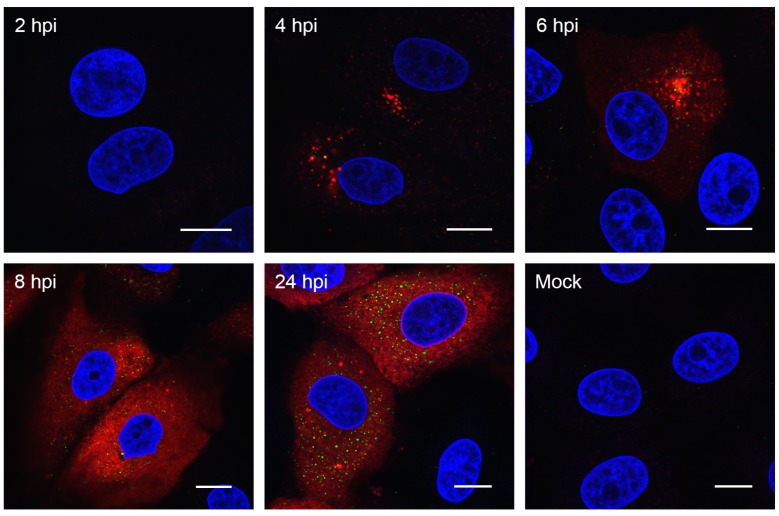
PDCoV-associated dsRNA can be detected from 4 h post-infection (hpi). LLC-PK1 cells were infected or mock-infected. At the stated timepoints, cells were fixed and labelled with anti-dsRNA (green) and anti-N (red). Nuclei were stained with DAPI (blue) and scale bar indicates 10 μm. Images are representative of three independent replicates.

To begin to characterize PDCoV RO, the cellular location of N protein and dsRNA was visualized. Coronavirus replication is associated with the accumulation of dsRNA in cytoplasmic puncta [20,24,39,41]. Although the precise nature and role of this dsRNA is debated, the location of dsRNA is regularly used as a marker for the sites of viral RNA synthesis. Due to the shortage of available reagents to visualize PDCoV proteins in infected cells and to allow comparison to the numerous previous studies on other coronaviruses, cells were infected or mock-infected and were fixed and labelled with antibodies specific for N and dsRNA at different timepoints following infection (Figure 2). No signal could be detected in mock-infected cells or in cells fixed at 2 hpi. However, at 4 hpi, clusters of cytoplasmic puncta of N protein were seen, with a smaller number of dsRNA puncta also visible. These appeared in the same area of the cell, but did not appear to co-localize. By 6 hpi, the number of both N and dsRNA puncta had increased. By 8 hpi, the N signal was found throughout the cytoplasm and appeared in a reticular staining pattern. The number of dsRNA puncta also continued to increase from 8 hpi to 24 hpi. These results indicate that viral RNA synthesis is likely to begin from around 4 hpi.

**Figure 3:**
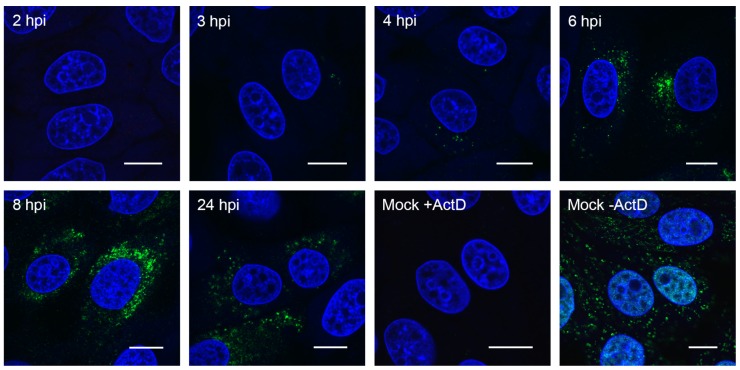
PDCoV RNA synthesis was detected from 3 hpi. LLC-PK1 cells were infected or mock-infected. Thirty minutes prior to the indicated fixation time, cells were incubated with 2 mM 5-Bromouridine (BrU) and 15 µM actinomycin D (ActD). Mock cells were incubated with (+ActD) and without (−ActD) actinomycin D as indicated. Cells were fixed and RNA containing BrU were detected using an anti-BrdU antibody (green). Nuclei were stained with DAPI (blue), scale bar indicates 10 μm. Images representative of three independent replicates.

To more conclusively determine the onset of viral RNA synthesis, as well as visualize where within the cell RNA synthesis is taking place, nascent RNA was labelled with 5-bromouridine (BrU). Cells were infected or mock-infected and were treated with BrU in the presence of actinomycin D (ActD) for 30 min prior to fixation to inhibit cellular RNA synthesis. BrU incorporated into nascent RNA was then detected using an anti-BrdU antibody (Figure 3). In mock-infected cells incubated in BrU without ActD, cellular RNA was detected in both the nucleus and cytoplasm, as expected. In the presence of ActD, this signal was lost. This confirmed that BrU signal detected in PDCoV infected cells was newly synthesized viral RNA. No BrU signal was detected at 2 hpi. However, at 3 hpi, individual cytoplasmic puncta or small clusters of puncta could be seen. The number of puncta increased as the infection progressed, and they became more widely dispersed throughout the cytoplasm. This demonstrates that PDCoV RNA synthesis begins from 2.5–3 hpi in small clusters within the cytoplasm and by later timepoints, numerous sites of RNA synthesis exist within the cell.

**Figure 4:**
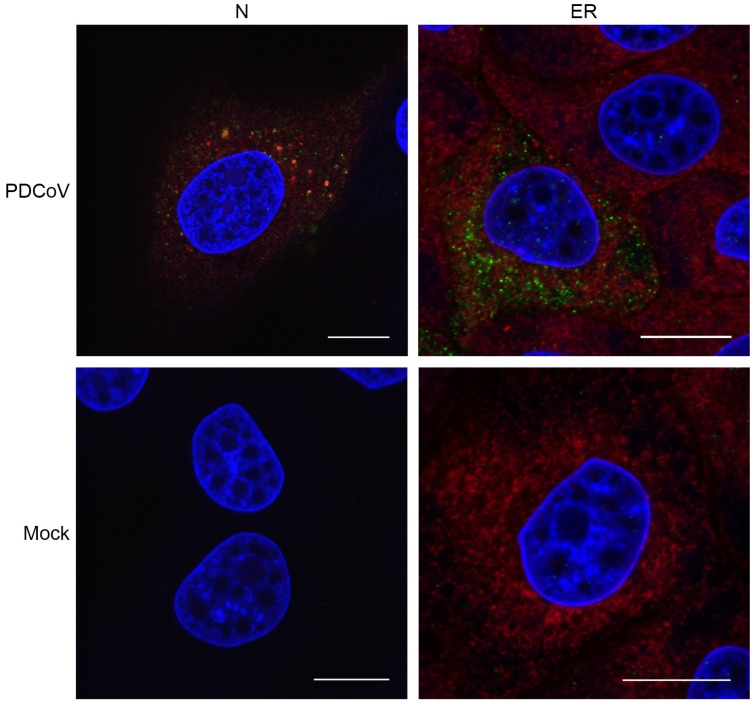
Sites of PDCoV RNA synthesis were associated with viral N protein, but not the endoplasmic reticulum (ER). LLC-PK1 cells were infected or mock-infected. Thirty minutes prior to fixation, cells were incubated with 2 mM 5-Bromouridine (BrU) and 15 μM actinomycin D. Cells were fixed and labelled with anti-BrdU (green) and either anti-N or anti-ER antibodies (red). Nuclei were stained with DAPI (blue), scale bar indicates 10 µm.

In order to gain more information about PDCoV ROs, the location of nascent viral RNA labelled with BrU was compared to the location of PDCoV N protein and an ER marker. The N protein binds to viral RNA forming the ribonucleoprotein complex, and genetic material is packaged into new particles in this form [42]. Furthermore, coronavirus ROs are connected to and contiguous with the cellular ER [20,24]. Therefore, nascent RNA could be expected to co-localize with these markers. Cells were infected or mock-infected with PDCoV. At 5.5 hpi, cells were treated with BrU and ActD as described previously, followed by fixation at 6 hpi and labelling to detect BrU, N, and ER (Figure 4). As seen before, BrU-labelled RNA was only detectable in PDCoV infected cells, not mock-infected cells, and the signal appeared in small cytoplasmic puncta. The BrU signal was found to be associated with the N protein, but it was not completely co-localized. Instead, the small BrU puncta appeared to localize near or next to larger N puncta (Figure 4). However, BrU puncta also appeared to exist that were not associated with N, and there were some N puncta that were not associated with BrU. When BrU localization was compared to an ER marker, there was no evidence of co-localization between the two signals.

### 3.3. PDCoV ROs Include Double-Membrane Vesicles and Zippered ER with Double-Membrane Spherules

**Figure 5:**
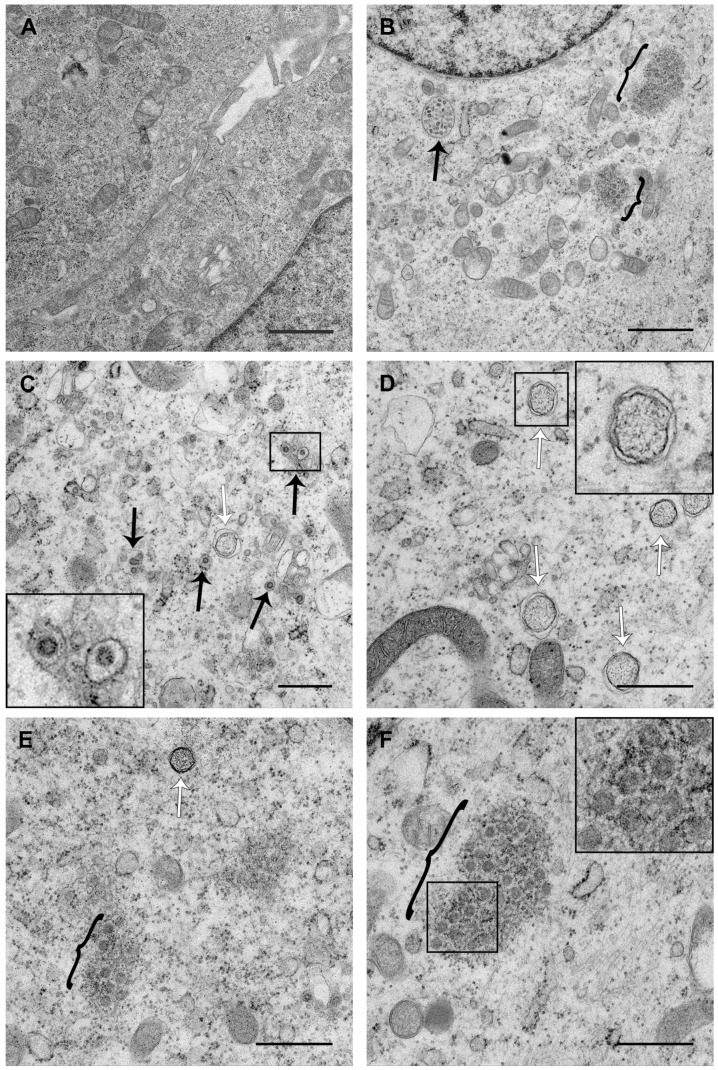
PDCoV RO is made up of double-membrane vesicles (DMVs) and zippered ER with double-membrane spherules. LLC-PK1 cells were mock-infected (**A**) or infected with PDCoV (**B**–**F**). At 8 hpi, cells were fixed with glutaraldehyde and processed for transmission electron microscopy. Virions in vesicles are indicated with black arrows, DMVs are indicated with white arrows, and regions of zippered ER with spherules are indicated with black brackets. Scale bars indicate 1 µm (**A**,**B**) or 500 nm (**C**–**F**).

It has recently been reported that PDCoV infection triggers the formation of double-membrane vesicles (DMVs) [36]. However, the presence of either CM or zippered ER and double-membrane spherules has not been demonstrated. Therefore, a detailed analysis of PDCoV ROs was performed. Cells infected with PDCoV or mock-infected were fixed at a range of timepoints post-infection and were embedded and processed for transmission electron microscopy analysis. Initially, samples from 8 hpi were imaged (Figure 5). At this timepoint, virus particles in vesicles were found, either as individual particles per vesicle (Figure 5C) or multiple particles in a single larger vesicle (Figure 5B). In addition, DMVs were clearly visible (Figure 5C–E). The samples were prepared by glutaraldehyde fixation and, as observed previously under these conditions, DMVs had a fibrous content and the two membranes were closely apposed in some areas, but had become separated from one another in other areas [21,43,44]. Large regions with numerous DMVs were observed, as well as both individual DMVs and clusters of small number of DMVs. In addition to DMVs, regions of small double-membrane vesicles were found interspersed with sections of paired membranes (Figure 5B,E,F). The small double-membrane vesicles had very tightly apposed membranes, and the membranes appeared to be lined with electron-dense content (zoomed in box, Figure 5F). The areas of paired membranes and small double-membrane vesicles were surrounded by electron-dense material and appeared highly comparable to regions of zippered ER with associated spherules identified previously in cells infected with *Gammacoronavirus* IBV [24]. Both large and small regions of zippered ER and spherules were observed in PDCoV infected cells, and they were most commonly found in the perinuclear region. Regions of zippered ER and spherules often, although not always, had a small number of DMVs in the vicinity. Together, this demonstrates that the PDCoV RO is made up of both DMVs and zippered ER with double-membrane spherules. 

### 3.4. PDCoV ROs, Including Zippered ER and Double-Membrane Spherules, are Visible from 6 hpi

**Figure 6:**
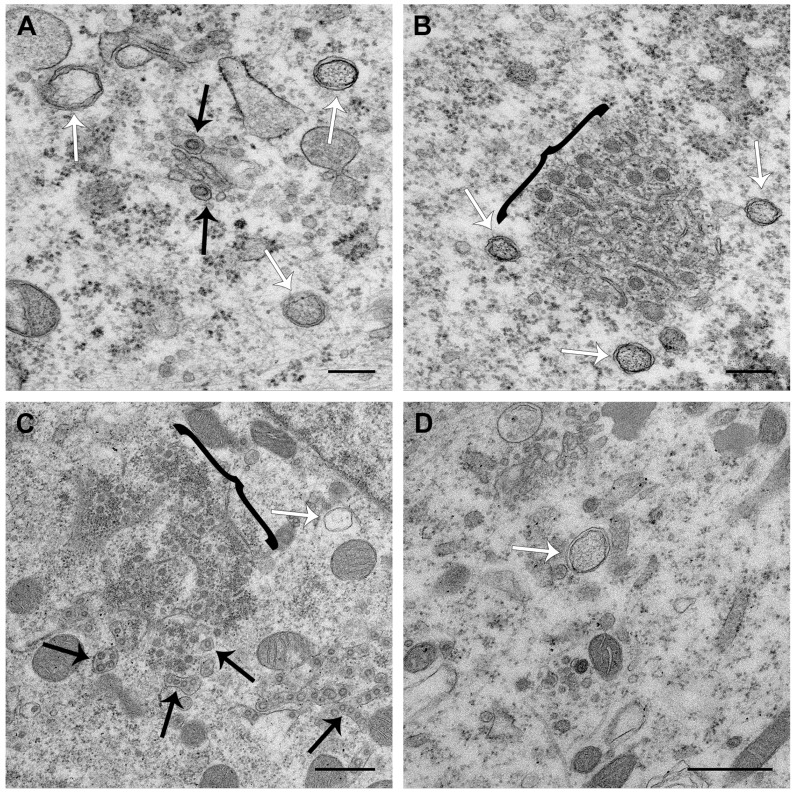
DMVs and zippered ER with double-membrane spherules were present from 6 hpi to 24 hpi. LLC-PK1 cells were infected with PDCoV, and at 6 hpi (**A**,**B**) or 24 hpi (**C**,**D**), cells were fixed with glutaraldehyde and processed for electron microscopy. Virions in vesicles are indicated with black arrows, DMVs are indicated with white arrows, and regions of zippered ER with spherules are indicated with black brackets. Scale bars indicate 200 nm (**A**) or 500 nm (**B**–**D**).

PDCoV RNA synthesis was detected in single puncta or small clusters of puncta from 3 hpi and in larger clusters of puncta from 4 hpi. By 6 hpi, the number of puncta had dramatically increased (Figure 3). Therefore, to further investigate whether ROs associated with this RNA synthesis could be detected earlier in infection than 8 hpi, a range of timepoints from 4–24 hpi were imaged (Figure 6). No ROs or any other evidence of virus infection were detected at 4 hpi. However, at 6 hpi and 24 hpi, ROs comprising both DMVs and regions of zippered ER and double-membrane spherules were found. ROs visualized at both 6 hpi and 24 hpi were highly comparable to those seen at 8 hpi. In addition, at 24 hpi, as well as virus particles in vesicles, virus particles were observed in the ER, presumably as a result of budding into the ER. Together, this confirms the presence of ROs made up of DMVs, zippered ER, and spherules from 6 hpi to 24 hpi, during the peak of PDCoV RNA synthesis.

## 4. Discussion

In this study, we characterized the replication dynamics of PDCoV OH-FD22 in porcine LLC-PK1 cells, with a particular focus on RO formation. Initial characterization of viral RNA, protein, and progeny production was performed. Although viral RNA could be detected from the start of the experiment at 2 hpi, this was most likely input genomic RNA. New RNA synthesis, as determined by an increase over this baseline level of RNA, was detected from 6 hpi, indicating that new viral RNA had been synthesized by this timepoint. This corresponded with detection of increased levels of viral protein at the same timepoint. The subsequent release of the progeny virus was detected from 10 hpi. This data is broadly consistent with observations for the replication dynamics of other coronaviruses, including MHV, SARS-CoV, IBV, and another lab-adapted strain of PDCoV [21,24,36,45,46,47].

Following the initial characterization of viral replication dynamics, the accumulation of virus replication associated dsRNA was visualized. Although historically used as a marker for sites of RNA synthesis, the role of dsRNA during coronavirus infection is not currently clear. It localized to the interior of DMVs in SARS-CoV infected cells [20], and although it co-localized with sites of active RNA synthesis at early timepoints in MHV infected cells, this was less obvious at later timepoints [39]. In addition, there was only partial co-localization between dsRNA and replicase proteins from other coronaviruses and more distantly related nidoviruses [5,20,21,43,44,48,49]. Therefore, it is not currently understood whether dsRNA represents a *bona fide* intermediate in viral RNA synthesis, is a byproduct of virus replication, or performs some other function. Despite this, dsRNA accumulation correlated with the onset of viral RNA synthesis and provides a useful marker, especially considering the limited availability of reagents to study PDCoV. Therefore, the accumulation of dsRNA in PDCoV-infected cells and co-localization with N protein was determined. Both dsRNA and N were detected in a small number of cytoplasmic puncta from 4 hpi, and these puncta were clustered together. From 6 hpi, the number of dsRNA puncta increased, and the puncta became more dispersed throughout the cytoplasm, as has been seen for other coronaviruses [20,24,39]. The number of N puncta also increased by 6 hpi, and from 8 hpi onward, the N signal predominantly showed a cytoplasmic reticular pattern. This demonstrates that the onset of PDCoV protein synthesis occurred prior to 4 hpi, which was earlier than detected by western blot. It is not surprising that small changes in N levels were less likely to be detected at the population level as determined by western blot than when imaging individual infected cells. Also, in agreement with previous observations, there appeared to be little co-localization between dsRNA and N at any timepoint studied here. The dynamics of dsRNA accumulation and location within the PDCoV infected cell were comparable to other previously studied coronaviruses. Numerous questions remain regarding the identity, involvement, and importance of dsRNA in the replication cycle of coronaviruses. However, these questions are beyond the scope of the current work and will form the basis of future studies.

To gain a more conclusive picture of the onset and localization of viral RNA synthesis, BrU incorporation into nascent viral RNA was adopted. This allowed the visualization of RNA synthesized within a defined period of time (here, 30 min prior to fixation of cells). Using this technique, the onset of viral RNA synthesis was shown to be between 2.5 hpi and 3 hpi. Again, this was significantly earlier than detected at the population level using RT-qPCR, reflecting the increased sensitivity of single cell analyses. The sites of viral RNA synthesis appeared in individual puncta or small clusters of puncta in the perinuclear region. As the infection proceeded, comparable to sites of dsRNA accumulation, the number of BrU puncta increased and became more dispersed throughout the cytoplasm. By 4 hpi, there was a small increase in the number of puncta, but the number and spread of puncta increased dramatically by 6 hpi. Therefore, a small number of ROs was expected at 3 hpi and 4 hpi. However, by 6 hpi, numerous ROs were present throughout large areas of the cytoplasm. The dramatic increase in the number of sites of RNA synthesis (BrU puncta) by 6 hpi correlates with the observed increase in accumulated viral RNA detected by RT-qPCR, indicating that RT-qPCR is not sufficiently sensitive to detect small changes in total RNA at earlier timepoints.

Further analysis of the cellular location of viral RNA synthesis was performed. BrU-labelled nascent RNA was found to be associated with the N protein. As the N protein is known to bind to viral RNA forming the ribonucleoprotein complex, this was expected. Interestingly, however, the two signals were not found to completely co-localize. Instead, smaller BrU puncta were located on or next to the larger N puncta. Although the significance of this is unknown, it is possible that when N is directly bound to viral RNA, the antibody is not able to gain access, meaning that only N in certain conformations is detected by this method. Conversely, N binding of BrU-labelled RNA may prevent access of the anti-BrdU antibody. In addition to areas where N and nascent RNA were associated, there were also BrU puncta with no obvious N signal in the vicinity. These puncta possibly reflect viral mRNAs that are not bound by N protein, or it may be possible that some other viral RNAs exist in locations where N binding does not occur. Finally, an N protein signal was observed without any associated BrU signal. The N protein is a highly abundant protein and is known to be multifunctional. In addition to binding genomic RNA, N plays a role in regulating gene expression, cell cycle progression, and apoptosis, and can alter the cellular cytoskeleton [50,51,52,53]. Next, the association of BrU-labelled nascent viral RNA with the cellular ER was investigated. Here, no evidence of association or co-localization were observed. It may appear surprising that ROs do not co-localize with the ER, since that they are known to be derived from and connected to the ER [20,24,54]. However, although previous observations using other coronaviruses have noted some co-localization between markers for ROs and the ER, this was not complete, as substantial amounts of viral marker did not localize to the ER. Furthermore, an ER marker was found to be absent from SARS-CoV DMV membranes [44,49,54]. Indeed, virus-induced modification of the ER is substantial, especially in the case of the tightly apposed membranes of both the zippered ER and DMVs. It is highly probable that this results in the exclusion of normal ER markers from these modified membrane structures.

Finally, the ultrastructure of PDCoV ROs was investigated. In agreement with previous studies on PDCoV and numerous other coronaviruses, DMVs were found in PDCoV-infected cells. Furthermore, the appearance of these DMVs was indistinguishable from DMVs found in other coronavirus infected cells when fixed with glutaraldehyde. DMVs were first detected at 6 hpi and continued to be detected until 24 hpi, which was the latest timepoint studied. Throughout infection, DMVs were observed as individual vesicles or in either small or large clusters. However, as seen for IBV [24], the DMVs were rarely in close proximity. This made it unlikely for DMVs to have connections and form part of an interconnected membrane network, as observed for SARS-CoV [20]. In addition, the large clusters of DMVs were not found to be associated with zippered ER and spherules, suggesting there may be functional differences between these structures. Finally, no likely intermediates in DMV formation were observed at any timepoint. Therefore, it remains to be determined precisely how DMVs are formed.

The most significant finding from this study is the presence of regions of zippered ER and associated double-membrane spherules in PDCoV infected cells in addition to DMVs. Furthermore, no regions of more branching CMs were identified. Zippered ER and spherules were detected from 6 hpi to 24 hpi, and their appearances did not alter over this time course. Throughout infection, both large and small areas of zippered ER and spherules were found. These were predominantly in the perinuclear region. Often, although not always, a small number of DMVs were found in the proximity. Significantly, the regions of zippered ER and spherules were highly comparable to those identified previously in IBV-infected cells [24]. Therefore, the ROs of *Gamma-* and *Deltacoronaviruses* appear to be conserved.

Despite the observed difference in the ROs induced by different coronavirus genera, the role of the different parts of the RO during coronavirus replication and the precise location of viral RNA synthesis on these membranes remain key questions in the field. Understanding the role of the different parts of the RO will shed light on the significance of the differences observed between the structures induced by the different coronavirus genera. Ultimately, understanding the function and formation of coronavirus ROs will provide useful information to allow control of this important virus family.

## Supplementary Materials

The following are available online at https://www.mdpi.com/1999-4915/11/11/1030/s1, Figure S1: Standard curve for absolute quantitation of PDCoV RNA copies by RT-qPCR.
