# Supplementary material for: The Porcine Deltacoronavirus Replication Organelle Comprises Double-Membrane Vesicles and Zippered Endoplasmic Reticulum with Double-Membrane Spherules
Source: Viruses. 2019 Nov 5;11(11):1030. doi: 10.3390/v11111030 (PMC6893519; doi:10.3390/v11111030)
Supplement: Supplementary file 1 [file viruses-11-01030-s001.zip › Supplementary Figure S1.docx]

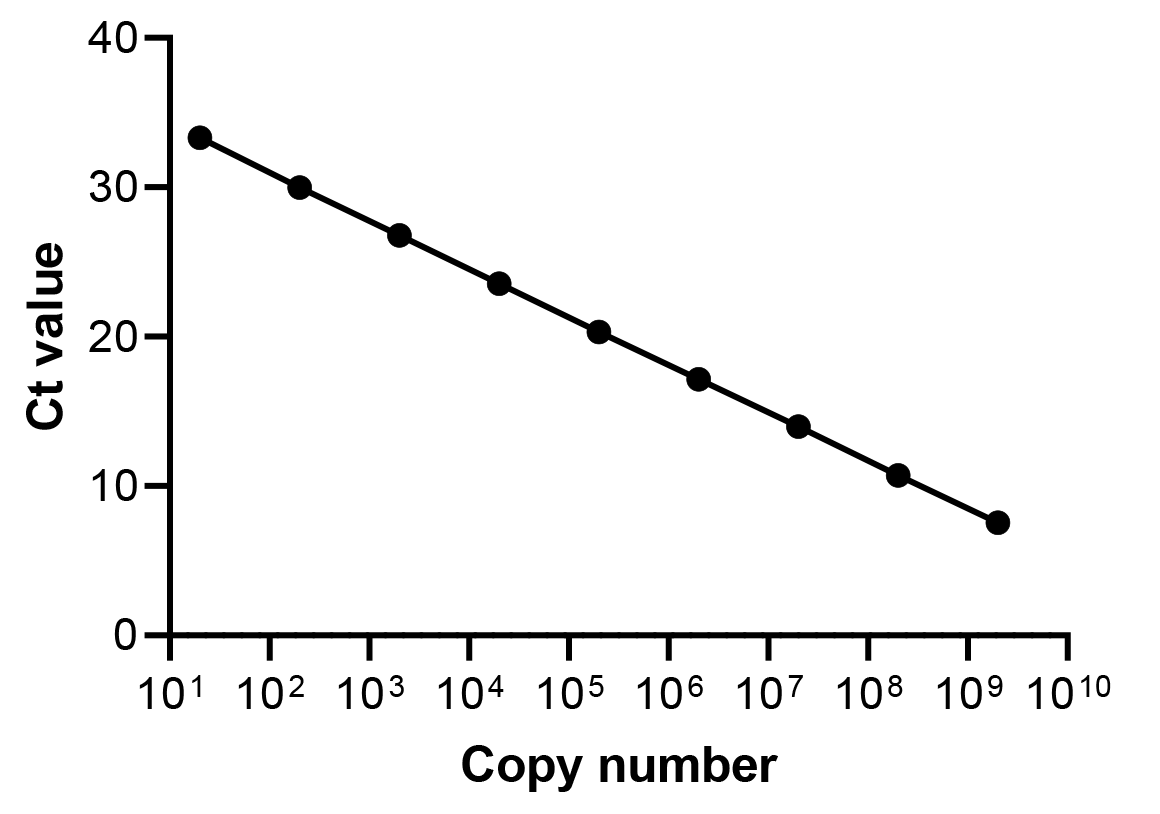


**Supplementary Figure S1.** Standard curve for absolute quantitation of PDCoV RNA copies by RT-qPCR. A series of known copy number standards of a PCR fragment containing the qPCR amplicon were included on each qPCR plate to allow absolute quantitation of the number of RNA copies present in the unknown samples.
