# Supplementary figures and images for: The Porcine Deltacoronavirus Replication Organelle Comprises Double-Membrane Vesicles and Zippered Endoplasmic Reticulum with Double-Membrane Spherules
Source: Viruses. 2019 Nov 5;11(11):1030. doi: 10.3390/v11111030 (PMC6893519; doi:10.3390/v11111030)

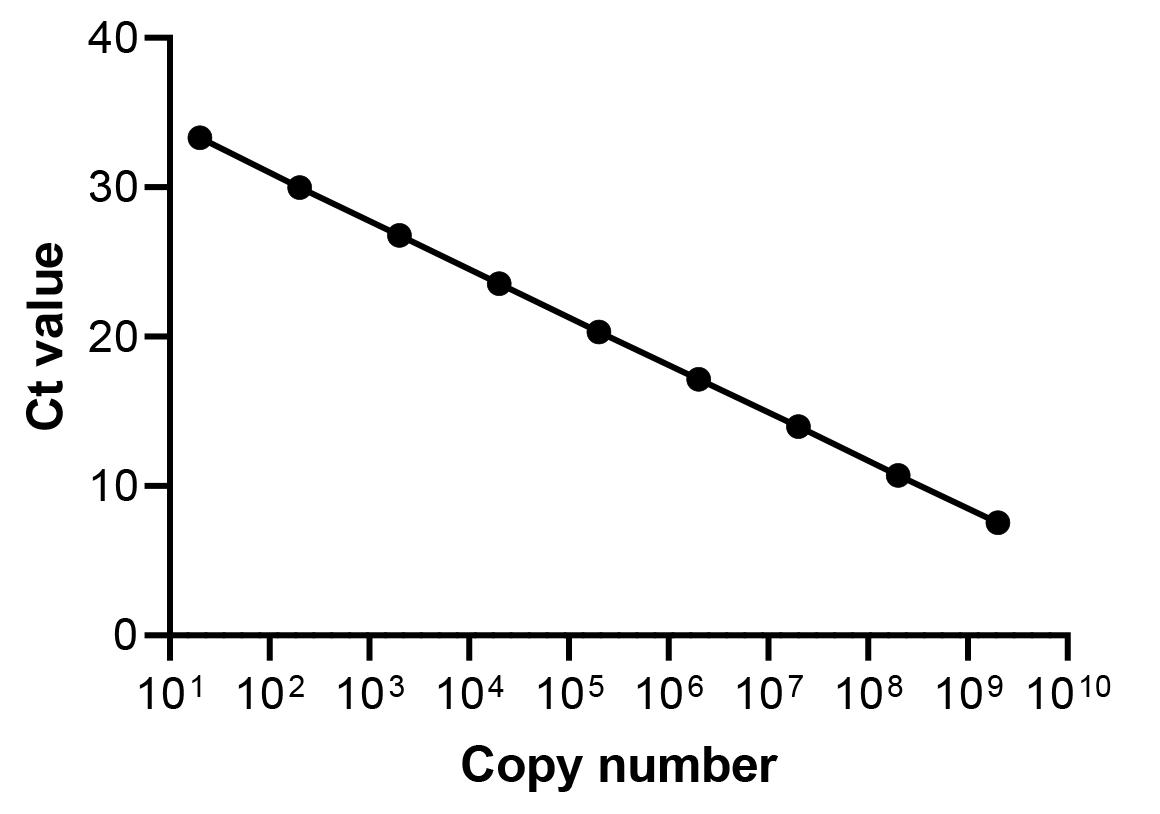

Supplement: Supplementary file 1 [file viruses-11-01030-s001.zip › Supp Fig 1.tif]
